# Microbial reference genomes for human metagenomics

**DOI:** 10.1186/gb-2011-12-s1-i7

**Published:** 2011-09-19

**Authors:** Sarah K Highlander

**Affiliations:** 1 Department of Molecular Virology and Microbiology, Baylor College of Medicine, Houston, TX 77030, USA

## 

A key resource for metagenomics projects is a representative catalog of annotated microbial genome sequences to serve as a reference for classification and functional annotation [1]. The Human Microbiome Project (HMP) [2], which is funded by the National Institutes of Health, began with the goal of sequencing 600 genomes from culturable prokaryotes that are representative of those inhabiting the major niches within and on the human body. This effort has been expanded to include uncultured organisms, small eukaryotes, and viruses that infect prokaryotes or eukaryotes, and the current sequencing target is 3,000 microbes. An automated pipeline for prokaryotic gene calling and annotation has been established for the project, yet very few of the HMP organisms have been subjected to in-depth analysis. Comparisons of several HMP genomes have revealed substantial intraspecies differences and provide clues about the pathogenic and physiological potential of these organisms.
